# Monomorphic xanthomatous type of xanthogranuloma: a rare entity

**DOI:** 10.3205/oc000070

**Published:** 2017-07-17

**Authors:** Pratheeba Devi Nivean, M. Nivean

**Affiliations:** 1 M.N. Eye Hospital, Chennai, India

**Keywords:** adult onset xanthogranuloma, chalazion, lid swellings

## Abstract

Xanthogranuloma is an uncommon tumor in the ocular adnexa. It usually presents as diffuse lid swelling with or without orbital involvement. Xanthogranulomas can be associated with systemic comorbidities. They are diagnosed by their characteristic pathological finding. They are further subclassified based on immunohistochemistry. We present two cases with lid swelling which were diagnosed to be xanthogranuloma. Our cases were special because of the unique presentation as localized monomorphic lesions.

## Introduction

Xanthogranulomatous diseases of the orbit are rare disorders of unknown etiology affecting the skin and subcutaneous tissues of the periorbital areas and the ocular adnexa [1]. Diagnosis is based on histopathology and immunohistochemistry. Orbital xanthogranulomatous disease has four subtypes: adult onset xanthogranuloma (AOX), necrobiotic xanthogranuloma (NBX), Erdheim-Chester disease (ECD), and adult onset asthma and periocular xanthogranuloma (AAPOX) [2], [3]. We report two cases of adult onset xanthogranuloma. The patients had small lid lesions which on histopathological evaluation was found to be xanthogranuloma. Our cases were unique as they were monomorphic and localized.

## Case description

### Case 1

**Figure 1:**
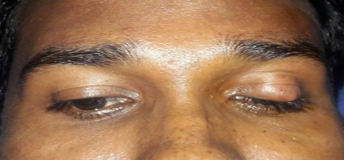
External photograph of the solitary lesion involving the left upper lid

**Figure 2:**
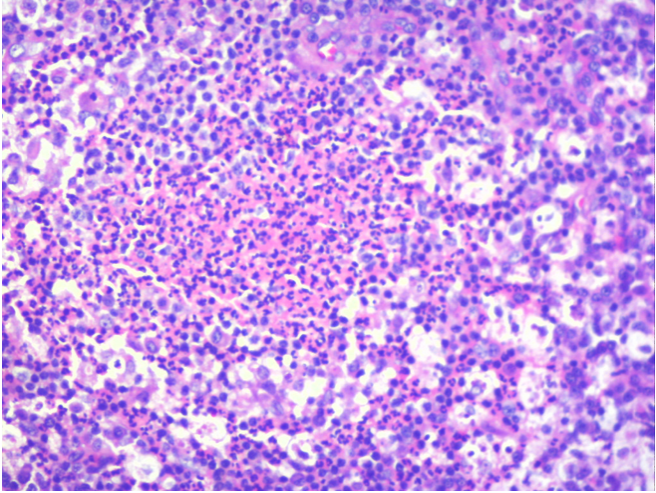
Histopathological examination showing polymorphs and neutrophilic microabscess

**Figure 3:**
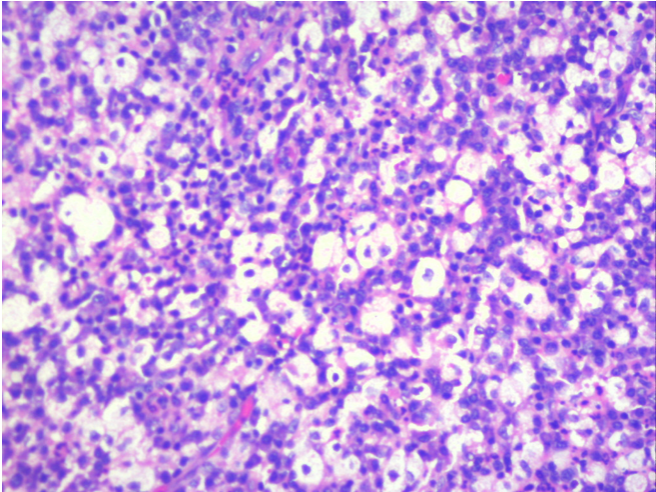
Histopathology showing foamy macrophages and plasma cells

**Figure 4:**
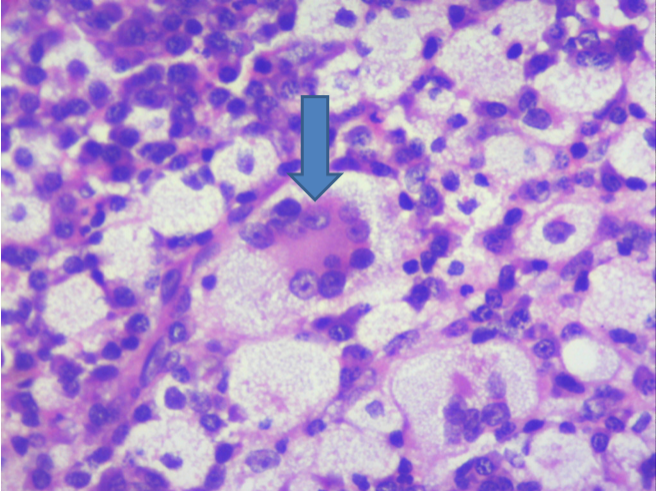
Histopathology showing Touton giant cell

A 36-year-old male came with complaints of left upper eyelid swelling since 1 year. It was painless, not progressive and was treated as chalazion elsewhere but did not resolve. On examination, his visual acuity was normal with correction. Intraocular pressure and extraocular movements were normal. The left eyelid showed a localized swelling confined to the tarsal region with a slight yellowish tinge seen over the skin (Figure 1 (Fig. 1)). There was no erythema or tenderness. There was no evidence of pus, loss of eyelashes, changes in lid margin or prominent vessels. The palpebral conjunctiva was normal. We suspected it to be a sebaceous cyst. Excision biopsy was done and the sample was sent for histopathological examination which revealed a mixture of cells including polymorphs, plasma cells (Figure 2 (Fig. 2)), foamy macrophages (Figure 3 (Fig. 3)), and occasional Touton type of giant cells (Figure 4 (Fig. 4)). The inclusions in the macrophages turned out to be PAS positive suggestive of xanthogranuloma. The patient's systemic parameters were normal and on his review after 12 months he was doing well without any recurrence. 

### Case 2

**Figure 5:**
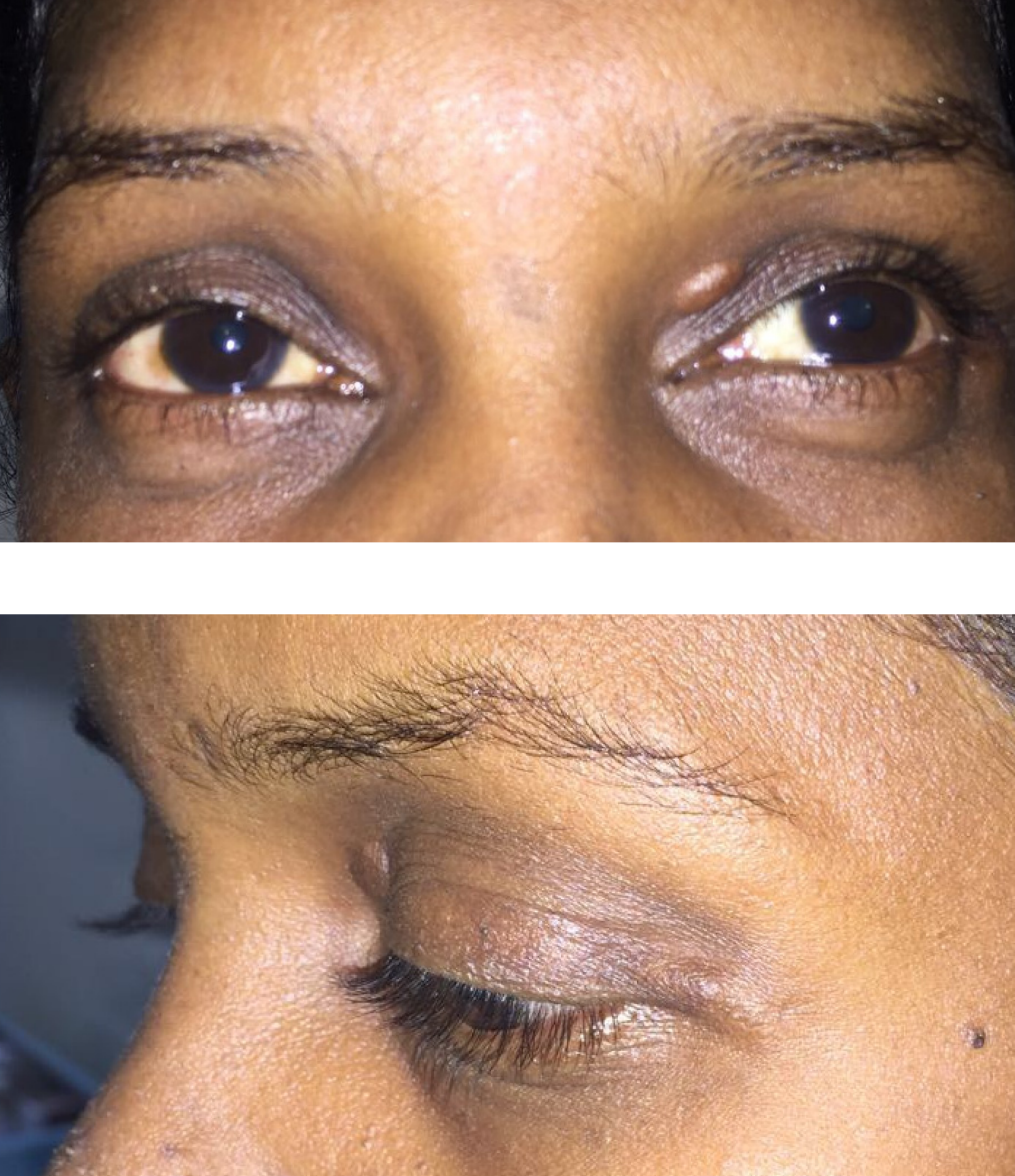
External photographs of the solitary lesion in the left eye

**Figure 6:**
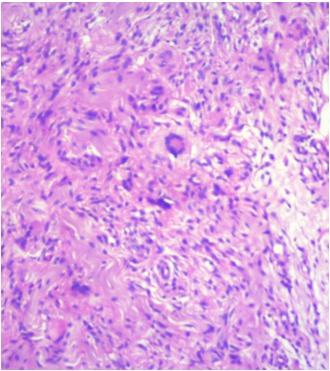
Histopathology showing mononucleated foamy histiocytes (xanthoma cells), aggregates of lymphocytes, plasma cells, and Touton giant cells

**Figure 7:**
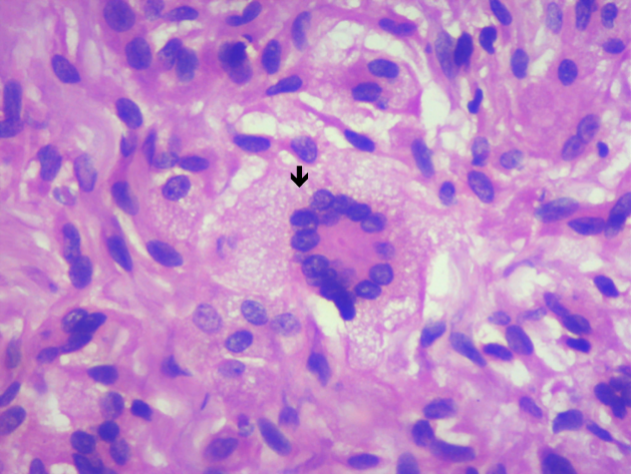
Magnified image (40x) showing the Touton cell

A 31-year-old female came with complaints of two pigmented painless lesions on her left upper eyelid since a few years (Figure 5 (Fig. 5)). The right eye was normal. The left upper eyelid showed two lesions with slight yellowish pigmentation on the medial and lateral side. Excision biopsy was advised which revealed plasma cells, touton cells and foamy histiocytes suggestive of a xanthogranulomatous inflammation (Figure 6 (Fig. 6), Figure 7 (Fig. 7)). We gave her a course of oral steroids. Physician evaluation was sought to rule out systemic involvement.

## Discussion

Adult onset xanthogranuloma though rare, is often missed in our clinical practice mostly due to its varied presentation and misdiagnosis. It could vary from a simple periorbital subcutaneous swelling to a serious disease involving multiple systems [4]. Most of the lesions of xanthogranuloma reported in literature are diffuse large swellings involving the lid and the orbit.

Our patient was previously treated for chalazion and if incision and curettage had been done instead of an excision biopsy, we too would have missed the diagnosis. The only clinical evidence was the yellowish tinge over the skin pointing towards xanthogranuloma (which we found retrospectively). We found our case different because none of the published cases of periocular xanthogranuloma was discrete and localized.

Juvenile xanthogranuloma (JXG) is a non-Langerhans type histiocytosis affecting the infants and young children and it is different from the adult onset xanthogranuloma. Ocular involvement is the most common extracutaneous manifestation of JXG whereas it is not present in AOX [5], [6].

Xanthogranuloma being a non-Langerhans histiocytosis could have systemic involvement such as eosinophilia, raised erythrocyte sedimentation rate and can involve the lungs, kidneys, trunk, and extremities [7], [8]. It could also be associated with Hyper-IgG4 syndrome. Systemic involvement is common in ECD. Adult onset xanthogranuloma is reported to have benign course and ECD has an aggressive course.

Though our first patient had no systemic involvement he should be monitored. Apart from routine evaluation serum electrophoresis and bone marrow biopsy to rule out multiple myeloma should be taken ideally. Our second patient had early onset hypertension and was on treatment. It could have been because of xanthogranulomatosis deposits in kidney (though we do not have any evidence). She also gave history of recurrent urinary tract infections, which again could be a feature of xanthogranuloma. Both our patients did not have asthma. They had small lesions in comparison to AOX wherein lesions were bulky involving the entire lid. Both the lesions had yellowish tinge over the lesion.

Histopathologically, the xanthogranuloma is characterized by sheets of mononucleated foamy histiocytes (xanthoma cells) infiltrating the orbital tissue, accompanied by aggregates of lymphocytes, plasma cells, and Touton giant cells. These infiltrating xanthoma cells stain with Oil Red O [9]. However this stain only works in unfixed material (cryostat sections) but not in formalin fixed and paraffin embedded tissue.

 Immunohistochemistry subclassifies the type of xanthogranuloma. Our patients had solitary and isolated lesions without asthma and systemic involvement so we diagnosed them to have AOX. 

Corticosteroids are the first line treatment in these lesions. But for recurrent and resistant lesions intralesional steroid injections have also been tried. Methotrexate could be an efficient corticosteroid sparing agent when symptoms recur [10], [11]. We did not give oral steroids to our first patient as the lesion was localized and it was removed in toto. Our patients had a follow-up of 1 year and he did not have any recurrence.

## Conclusion

As ophthalmologists, we must think of a broader differential diagnosis for even a simple eyelid swelling as not all will turn out to be a chalazion. Performing an excision biopsy in such cases gives us an accurate diagnosis and a thorough systemic examination helps in finding possible associations. Presence of yellowish hue over the lesion gives clue towards xanthogranuloma. These patients should be monitored for systemic involvement in future.

## Notes

### Competing interests

The authors declare that they have no competing interests.
